# Granulocyte and Monocyte Adsorption Therapy in Patients With Sepsis: A Feasibility Study

**DOI:** 10.1111/aor.14943

**Published:** 2025-01-17

**Authors:** Osamu Nishida, Tomoyuki Nakamura, Takaaki Nakada, Gaku Takahashi, Yoshiki Masuda, Hiroki Tsubouchi, Yasuyuki Kakihana, Yuichiro Sakamoto, Osamu Takasu, Hiroyuki Suzuki, Koichi Nakazawa, Iwao Kobayashi, Kent Doi, Sohta Uchiyama, Nobuya Kitamura, Toru Kotani, Naohide Kuriyama, Noriyuki Hattori, Yasushi Suzuki, Hiroomi Tatsumi, Kazuhiro Moriyama

**Affiliations:** ^1^ Department of Anesthesiology and Critical Care Medicine Fujita Health University School of Medicine Toyoake Japan; ^2^ Department of Emergency and Critical Care Medicine Chiba University Chiba Japan; ^3^ Department of Critical Care and Emergency Iwate Prefectural Advanced Critical Care and Emergency Center, Iwate Medical University Shiwa Japan; ^4^ Department of Intensive Care Medicine Sapporo Medical University School of Medicine Sapporo Japan; ^5^ Department of Anesthesiology and Intensive Care Ichinomiyanishi Hospital Ichinomiya Japan; ^6^ Department of Emergency and Intensive Care Medicine Kagoshima University Graduate School of Medical and Dental Sciences Kagoshima Japan; ^7^ Department of Emergency and Critical Care Medicine Saga University, Saga Saga Japan; ^8^ Department of Emergency and Critical Care Medicine Kurume University Kurume Japan; ^9^ Advanced Medical Emergency Department and Critical Care Center Japanese Red Cross Maebashi Hospital Maebashi Japan; ^10^ Department of Anesthesiology Tokyo Medical University Shinjuku Japan; ^11^ Critical Care and Emergency Center Japanese Red Cross Asahikawa Hospital Asahikawa Japan; ^12^ The Department of Emergency and Critical Care Medicine The University of Tokyo Tokyo Japan; ^13^ Japan Department of Anesthesiology and Intensive Care Medicine Nishichita General Hospital Tokai Japan; ^14^ Emergency and Critical Care Center Kimitsu Chuo Hospital Kisaraz Japan; ^15^ Department of Intensive Care Medicine Showa University School of Medicine Shinagawa Japan; ^16^ Laboratory for Immune Response and Regulatory Medicine Fujita Health University School of Medicine Toyoake Japan

**Keywords:** adacolumn, cytokine, hemoadsorption, neutrophils, sepsis, sequential organ failure assessment

## Abstract

**Background:**

The pathogenesis of sepsis is thought to be linked to a dysregulated immune response, particularly that involving neutrophils. We have developed a granulocyte adsorption column as a “decoy organ,” which relocates the massive inflammation in organs in the body to a blood purification column. This study was conducted to assess the safety and experimental effectiveness of granulocyte monocyte adsorption apheresis‐direct hemoperfusion (G1‐DHP) in the treatment of patients with sepsis, using a prospective, multicenter design.

**Methods:**

The study included patients diagnosed with sepsis and with an APACHE II score ranging from 17 to 34. A total of five G1‐DHP were performed within 3 days of patient enrollment. The primary endpoint was the change in sequential organ failure assessment (SOFA) score from enrollment to 7 days, and the safety endpoints were adverse events and mortality at 28 days.

**Results:**

G1‐DHP was performed on 82 patients. The median (interquartile range) SOFA score decreased from 10 (8–11) to 4 (3–7) after 7 days (*n* = 70; *p* < 0.01). Granulocytes, mainly neutrophils, were adsorbed, and the neutrophil‐to‐lymphocyte ratio significantly improved (*p* < 0.01). Notable improvements were observed in the SOFA scores for circulation and renal function. The acute physiology and chronic health evaluation II score of the 77 patients evaluated for mortality was 27, and the 28‐day mortality rate was 7.8%.

**Conclusions:**

This study confirmed that G1‐DHP can be safely used as an adjunct to standard sepsis treatment regimens. Although further investigations are required, G1‐DHP is a promising supplemental therapy for sepsis.

**Trial Registration:** jRCT1080225183 (Japan Registry of Clinical Trials identifier)

## Graphical abstract

The median SOFA score decreased from 10 to 4 after 7 days. Decreased circulatory and renal SOFA scores were characteristic. Granulocytes, mainly neutrophils, were adsorbed, and the neutrophil‐to‐lymphocyte ratio significantly improved. The median APACHE II score was 27, and the 28‐day mortality rate was 7.8%.

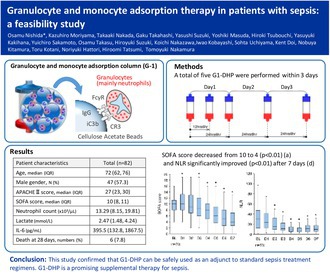

## 1 Introduction

Sepsis remains a leading cause of death in the intensive care unit (ICU). Despite recent advances in ICU and sepsis management, mortality rates remain high. Approximately 11 million deaths occur annually due to sepsis, accounting for approximately 20% of deaths worldwide [1]. The pathophysiology of sepsis involves many complex cellular and biochemical interactions between leukocytes, platelets, endothelial cells, and the complement system, which trigger an inflammatory response [2]. However, as the definition of sepsis indicates, the main players in the dysregulated host response during sepsis are immune cells, such as neutrophils, and the mediators produced by these cells [3]. Extracorporeal blood purification techniques have been proposed as adjunct therapies for sepsis. These techniques are based on the principle that removal of bacterial toxins, such as endotoxin, and inflammatory mediators can attenuate the sepsis‐related massive systemic inflammatory response, reducing morbidity and mortality. The most extensively studied therapy is endotoxin adsorption using a polymyxin B‐immobilized fiber column (PMX) [4]. Other reported blood purification devices include cytokine adsorption columns [5] and hemofilters [6, 7]. Despite the availability of blood purification methods, evidence of their therapeutic efficacy is limited.

For this reason, treatment of sepsis is considered insufficient to counteract mediators, and the idea of targeting immune cells has also been suggested [8, 9]. Neutrophils, the mainstay of innate immunity, have an extended lifespan during sepsis [10] and circulate in the bloodstream for long periods, resulting in persistent tissue damage and other undesirable effects [11]. Therefore, we hypothesized that blood purification using a granulocyte and monocyte adsorption column (G‐1: equivalent to the commercially available Adacolumn, JIMRO Co. Ltd., Gunma) would be effective for the treatment of sepsis. The concept of G‐1 direct hemoperfusion (G1‐DHP) involves using a granulocyte and monocyte adsorption column as a “decoy organ”. This approach is intended to relocate the massive systemic inflammation in the organs in a body to the “Adacolumn decoy organ”. Accordingly, the neutrophil activity in the blood is expected to weaken, and as a result, organ damage could be prevented [12]. We have confirmed and reported the effects of Adacolumn on granulocytes and monocytes during sepsis in ex vivo [13] and animal studies [14]. This study aimed to evaluate the safety and pilot efficacy of G1‐DHP, in addition to standard therapy, in patients with sepsis admitted to the ICU.

## 2 Patients and Methods

### 2.1 Study Design

This was a prospective, multicenter, single‐clinical trial. The study protocol was approved by the ethics committee of each participating institution and was conducted according to the guidelines set by the relevant institutional review boards. While conducting this study, we adhered to the principles of the Declaration of Helsinki and the Good Clinical Practice guidelines. All patients or their legally authorized representatives were provided written informed consent before participating in the study.

### 2.2 Patient Selection

Patients with sepsis were identified according to predefined inclusion criteria and enrolled in this study within 24 h of admission to the ICU. The participants were patients who met all the following criteria: (1) diagnosed with sepsis according to the Sepsis‐3 definition [3], (2) aged between 18 and 85 years, and (3) acute physiology and chronic health evaluation (APACHE) II score of 17–34 [15]. The following patients were excluded: (1) patients predicted to die within 3 days, (2) patients who had not undergone surgical procedures for removal of infection foci, (3) patients who had an organ transplantation within the previous year, (4) patients suspected of having human immunodeficiency virus or human T‐lymphotropic virus type I infection, (5) patients receiving long‐term treatment for immunodeficiency, (6) patients determined to have an increased tendency to hemorrhage due to the use of G‐1, (7) patients with a white blood cell count < 4000 cells/mm^3^, and 8) patients who had received extracorporeal membrane oxygenation. No treatment limitations, which hindered the standard treatment of sepsis, were imposed.

### 2.3 Procedure for G‐1 Administration

G1‐DHP was initiated within 3 h of patient enrollment. Each use was generally set at a blood flow rate of 50 mL/min for 120 min (the processed blood volume was 6000 mL), with a guideline to use five columns within 3 days. The second round commenced 12 h (±6 h) after the first round, the third round commenced 24 h (±6 h) after the first round, and the fourth and fifth rounds commenced 24 h (±6 h) after the previous use.

### 2.4 Data Collection

After obtaining informed consent, data on age, sex, APACHE II score, infection site, treatment of sepsis, and renal replacement therapy during G1‐DHP were collected. Clinical data were recorded at baseline, Day 3, and Day 7 after enrollment. This included the sequential organ failure assessment (SOFA) score, serum levels of lactate, interleukin (IL)‐6, IL‐8, IL‐10, high mobility group box‐1 protein (HMGB‐1), C‐reactive protein, fibrin degradation products (FDP), and D‐dimer. Urine neutrophil gelatinase‐associated lipocalin (NGAL) level, hourly urine volume, biochemical tests for the calculation of the SOFA score, complete blood count, neutrophil‐to‐lymphocyte ratio (NLR), severity classification of acute kidney injury (AKI) (Kidney Disease: Improving Global Outcomes (KDIGO) stage) [16], the disseminated intravascular coagulation (DIC) score based on the diagnostic criteria for acute DIC [17], the ratio of partial arterial pressure of oxygen to fractional inspired concentration of oxygen (PaO_2_/F_I_O_2_), mean arterial pressure (MAP), and the norepinephrine equivalent vasopressor dosage were also collected [18]. Urinary NGAL and HMGB‐1 levels were measured at baseline and Day 7. The number of ventilator‐free days at 28 days after ICU admission, the number of ICU stay days until 28 days after ICU admission, and the 28‐days outcome were recorded.

### 2.5 Endpoint

The primary efficacy endpoint was the change in the SOFA score (delta SOFA score) in surviving patients at Day 7 after enrollment, with a target value of a reduction ≤ 2 points. The primary safety endpoint was 28‐day mortality after enrollment.

Secondary endpoints included the adsorption rate and number of white blood cells on the G1 adsorbent in the first round of G1‐DHP, changes over 7 days in the number of neutrophils, lymphocytes, and the NLR; organ‐specific SOFA scores; various organ‐specific indicators; lactate level; serum cytokine levels; temporal changes in hemodynamics; and incidence of adverse events. To evaluate organ‐specific SOFA scores, the influence of continuous renal replacement therapy (CRRT) was also considered, and the scores were evaluated based on the presence or absence of concomitant CRRT.

Hemodynamics were evaluated using MAP and the norepinephrine‐equivalent vasopressor dosage, and renal function was evaluated using changes in the KDIGO stage, serum creatinine, urine volume, and urinary NGAL level. The progression of stages was also confirmed, as the survival rate decreased according to severity (stages 1, 2, and 3) [19]. As baseline urine volume data were not always available, the change in hourly urine volume from Day 1 to Day 7 was evaluated. Coagulation was evaluated using the DIC score, which is calculated based on the presence or absence of systemic inflammatory response syndrome, number and degree of decrease in platelets, prothrombin time ratio, and FDP. The respiratory function was evaluated using PaO_2_/F_I_O_2_. Changes in lactate, serum cytokines, FDP, and D‐dimer levels were evaluated by dividing patients into high and low groups based on baseline values. In addition, an increase in the number of patients with a high lactate level and DIC complications was confirmed. Additionally, ventilator‐free days at 28 days (non‐survivors were counted by date of death) and days in the ICU (non‐survivors were counted at 28 days) were recorded.

### 2.6 Statistical Analysis

Data were summarized using descriptive statistics according to their distribution (normal or non‐normal), and an appropriate testing method was selected. Analytical results are expressed as mean ± standard deviation or median (interquartile range). Primarily, the Wilcoxon signed‐rank test was used to compare parameters relative to the baseline values. Statistical significance was set at *p* < 0.05. Data analysis was performed using JMP 12.0.1 (SAS Institute Inc., Cary, North Carolina, USA).

## 3 Results

### 3.1 Feasibility

This study included 83 patients from 15 hospitals in Japan, and G1‐DHP was performed on 82 patients. Medical staff, including ICU doctors, clinical engineers, and nurses, were able to perform G1‐DHP without any complications, and there were no other adverse technical events. G1‐DHP was performed in combination with other renal replacement therapies in 52.4% of the patients, and no problems were reported. There was one case in which clotting was observed inside the column during blood return; however, it was considered to be derived from the patient rather than from a product defect, and there were no adverse events. The platelet count decreased from 118.5 × 10^3^/μL (56.8 × 10^3^/μL–162.5 × 10^3^/μL) at baseline to 58.5 × 10^3^/μL (35.0 × 10^3^/μL–107.3 × 10^3^/μL) on Day 3; however, it was 154.0 × 10^3^/μL (84.0 × 10^3^/μL–244.8 × 10^3^/μL) on Day 7, which was significantly higher than that at admission to the ICU (*p* < 0.01) (Table 1). Serious adverse events were reported in 10 (12.2%) patients. Among them, pneumonia, acute cholecystitis, and anaphylactic reactions in three (3.7%) patients could not be ruled out as being related to the G1‐DHP.

**TABLE 1:** Outcomes from baseline to Day 3 and Day 7.

	*N*	Baseline	Day 3	Day 7	p*	p**
SOFA score, median (IQR)	70	10 (8, 11)	9 (6, 12)	4 (3, 7)	< 0.05	< 0.01
ΔSOFA	70	—	−1 (−4, 2)	−5 (−7, −3)		
Organ‐specific SOFA score, mean ± SD						
Central nervous system	70	1.47 ± 1.43	1.36 ± 1.31	0.91 ± 1.21	n.s.	< 0.01
Respiratory	70	1.40 ± 1.07	1.31 ± 0.93	1.04 ± 0.81	n.s.	< 0.05
Cardiovascular	70	3.31 ± 1.06	1.63 ± 1.63	0.53 ± 1.06	< 0.01	< 0.01
Liver	70	0.71 ± 0.93	0.76 ± 0.86	0.59 ± 0.91	n.s.	n.s.
Kidney	70	1.63 ± 1.30	1.79 ± 1.55	1.01 ± 1.38	n.s.	< 0.01
Coagulation	70	1.23 ± 1.17	2.06 ± 1.15	0.93 ± 1.15	< 0.01	< 0.05
Specific organ damaged indicators, median (IQR)						
KDIGO stage	70	3 (2, 3)	3 (0, 3)	1 (0, 3)	< 0.01	< 0.01
Acute DIC score	70	4 (3, 5)	4 (2, 5)	2 (1, 4)	n.s.	< 0.01
PaO_2_/FiO_2_	70	284.0 (180.8, 400.8)	330.3 (233.2, 379.8)	363.3 (287.7, 410.6)	n.s.	< 0.01
MAP (mmHg)	70	78.35 (67.70, 84.70)	81.00 (70.70, 94.78)	83.15 (74.23, 93.25)	< 0.05	< 0.01
Norepinephrine equivalent vasopressor dosage (μg/kg/min)	64	0.089 (0.039, 0.163)	0.023 (0.000, 0.117)	0.000 (0.000, 0.000)	< 0.01	< 0.01
Urine volume (mL/h)^a^	66	62.56 (25.58, 121.09)	96.29 (47.29, 128.23)	69.35 (38.93, 93.14)	n.s.	n.s.
Creatinine (mg/dL)	70	1.67 (1.18, 2.91)	0.85 (0.64, 1.32)	0.90 (0.59, 1.56)	< 0.01	< 0.01
Total bilirubin (mg/dL)	70	1.15 (0.60, 1.63)	1.15 (0.70, 1.98)	0.90 (0.50, 1.63)	n.s.	< 0.05
Blood count, median (IQR)						
Neutrophil count (×10^3^/μL)	69	13.58 (8.53, 20.11)	9.05 (6.44, 12.37)	9.34 (6.44, 12.93)	< 0.01	< 0.01
Lymphocyte count (×10/μL)	68	0.58 (0.22, 0.85)	0.73 (0.47, 1.15)	0.91 (0.54, 1.24)	< 0.01	< 0.01
Platelet count (×10^3^/μL)	70	118.5 (56.8, 162.5)	58.5 (35.0, 107.3)	154.0 (84.0, 244.8)	< 0.01	< 0.01
Lactate (mmol/L)	58	2.60 (1.47, 4.41)	1.30 (0.95, 1.91)	1.00 (0.86, 1.31)	< 0.01	< 0.01
Urinary NGAL (ng/mL)	65	782.0 (180.5, 3745.0)	—	163.0 (52.7, 1110.0)	—	< 0.01
CRP (mg/dL)	70	16.02 (9.22, 24.98)	8.59 (5.26, 13.09)	5.53 (2.78, 8.42)	< 0.01	< 0.01
FDP	70	20.60 (11.03, 51.08)	15.35 (9.80, 27.43)	14.30 (8.68, 26.50)	< 0.05	< 0.01
D‐dimer (μg/mL)	70	10.10 (4.65, 22.03)	7.85 (4.65, 17.92)	7.20 (3.72, 13.80)	n.s.	< 0.05

*Note:* Analysis was conducted in a patient group that received G‐1 direct hemoperfusion at least once and for whom efficacy evaluation was possible. Data are expressed as medians (Interquartile ranges) for continuous variables, except for organ‐specific SOFA scores, which are presented as mean ± SD, and numbers (%) for categorical variables. *P* values were calculated using the Wilcoxon signed‐rank test (*: Baseline vs. Day 3, **: Baseline vs. Day 7).

Abbreviations: MAP, mean arterial pressure; SOFA, sequential organ failure assessment.

^a^ Only urinary volume (mL/h) was analyzed using data at Day 1 instead of baseline data because there many missing data at baseline.

### 3.2 Patient Characteristics

The baseline characteristics of the patients are shown in Table 2. The patients included 47 men (57.3%) and 35 women (42.7%), with a median age of 72 (62–76) years, and an APACHE II score of 27 points (23–30). Seventy‐one patients achieved 28‐day survival, and six patients died (7.8%). There was no difference in severity or background at baseline between survivors and non‐survivors. Although PMX‐DHP was more commonly used in non‐survivors, no differences were observed in other treatment conditions between survivors and non‐survivors.

**TABLE 2:** Patient characteristics.

	Total (*n* = 82)	Survivors (*n* = 71)	Non‐survivors (*n* = 6)	*p*
Age, median (IQR)	72 (62, 76)	72 (62, 76)	75 (68, 80)	n.s.
Male gender, *N* (%)	47 (57.3)	40 (56.3)	3 (50.0)	n.s.
APACHE II score, median (IQR)	27 (23, 30)	27 (23, 30)	29 (25, 34)	n.s.
SOFA score, median (IQR)	10 (8, 11)	10 (8, 11)	10 (9, 12)	n.s.
Causative microorganisms, *N* (%)				
Gram‐positive coccus	54 (65.9)	51 (71.8)	3 (50.0)	n.s.
Gram‐negative bacillus	62 (75.6)	57 (80.3)	4 (66.7)	n.s.
Fungus	36 (43.9)	32 (45.1)	4 (66.7)	n.s.
Infection site, *N* (%)				
Respiratory	14 (17.1)	11 (15.5)	2 (33.3)	n.s.
Gastrointestinal	29 (35.4)	27 (38.0)	1 (16.7)	n.s.
Renal and urinary	23 (28.0)	21 (29.6)	1 (16.7)	n.s.
Other	25 (30.5)	21 (29.6)	2 (33.3)	n.s.
Ingrammation‐related tests, median (IQR)				
Neutrophil count (×10^3^/μL)	13.29 (8.15, 19.81)	13.41 (8.01, 20.06)	10.63 (7.25, 20.81)	n.s.
Lactate (mmol/L)	2.47 (1.48, 4.24)	2.80 (1.55, 4.35)	1.80 (1.10, 6.13)	n.s.
IL‐6 (pg/mL)	395.5 (132.8, 1867.5)	376.0 (132.0, 1920.0)	841.5 (81.8, 5212.5)	n.s.
CRP (mg/dL)	16.79 (9.22, 25.30)	17.01 (9.22, 25.30)	15.60 (9.82, 25.56)	n.s.
Acute kidney injury (AKI), *N* (%)	73 (89.0)	63 (88.7)	5 (83.3)	n.s.
Disseminated intravascular coagulation (DIC), *N* (%)	48 (58.5)	45 (63.4)	2 (33.3)	n.s.
Treatment, *N* (%)				
Antibiotics	82 (100.0)	71 (100.0)	6 (100.0)	—
Inotrope or vasopressors	74 (90.2)	63 (88.7)	6 (100.0)	n.s.
Sterodis	53 (64.6)	46 (64.8)	5 (83.3)	n.s.
rTM/AT III	63 (76.8)	57 (80.3)	5 (83.3)	n.s.
Mechanical ventilation, *N* (%)	59 (72.0)	50 (70.4)	5 (83.3)	n.s.
Renal replacement therapy during G1‐DHP, *N* (%)				
Any RRT use	43 (52.4)	39 (54.9)	3 (50.0)	n.s.
PMX‐DHP	3 (3.7)	1 (1.4)	2 (33.3)	< 0.05
CRRT	34 (41.5)	31 (43.7)	2 (33.3)	n.s.
IRRT	15 (18.3)	15 (21.1)	0 (0.0)	U.S.
No RRT use	39 (47.6)	32 (45.1)	3 (50.0)	n.s.
Ventilator‐free days, median (IQR)	23 (13, 25)	—	—	—
ICU length of stay, median (IQR)	9 (7, 16)	—	—	—
Death at 28 days, median (IQR)	6 (7.8)			

*Note:* The data shown is from the patient group that started using G‐1 direct hemoperfusion. Data are expressed as medians (Interquartile ranges) for continuous variables and numbers (%) for categorical variables. The Mann–Whitney *U* test was used for the continuous variable, and the Chi‐square test was used for categorical variables. Acute kidney injury (AKI) applies to patients with a renal function score of 1 to 3 at baseline. Disseminated intravascular coagulation (DIC) applies to patients with an acute phase DIC diagnostic score of 4 or more at baseline. APACHE II: acute physiology and chronic health evaluation II; SOFA: sequential organ failure assessment; rTM: recombinant human soluble thrombomodulin; ATIII: antithrombin III; RRT: renal replacement therapy, CRRT: continuous renal replacement therapy; IRRT: intermittent renal replacement therapy, PMX‐DHP: polymyxin B‐immobilized fiber column‐direct hemoperfusion, n.s.: not significant.

### 3.3 G1‐DHP Implementation Status

Out of 82 patients, 78 completed at least one round of G1‐DHP, and 57 patients (69.5%) completed five rounds, with a mean of 4.4 ± 1.20 rounds. The anticoagulants used in the G1‐DHP procedures were nafamostat mesilate in 77 patients (93.9%) and heparin in 4 patients (4.9%). There was one patient (1.2%) in whom no anticoagulant was used because of prolonged activated partial thromboplastin time. Five patients discontinued participation.

### 3.4 Evaluation Results

#### 3.4.1 The Primary Efficacy Endpoint: Delta SOFA Score

**FIGURE 1:**
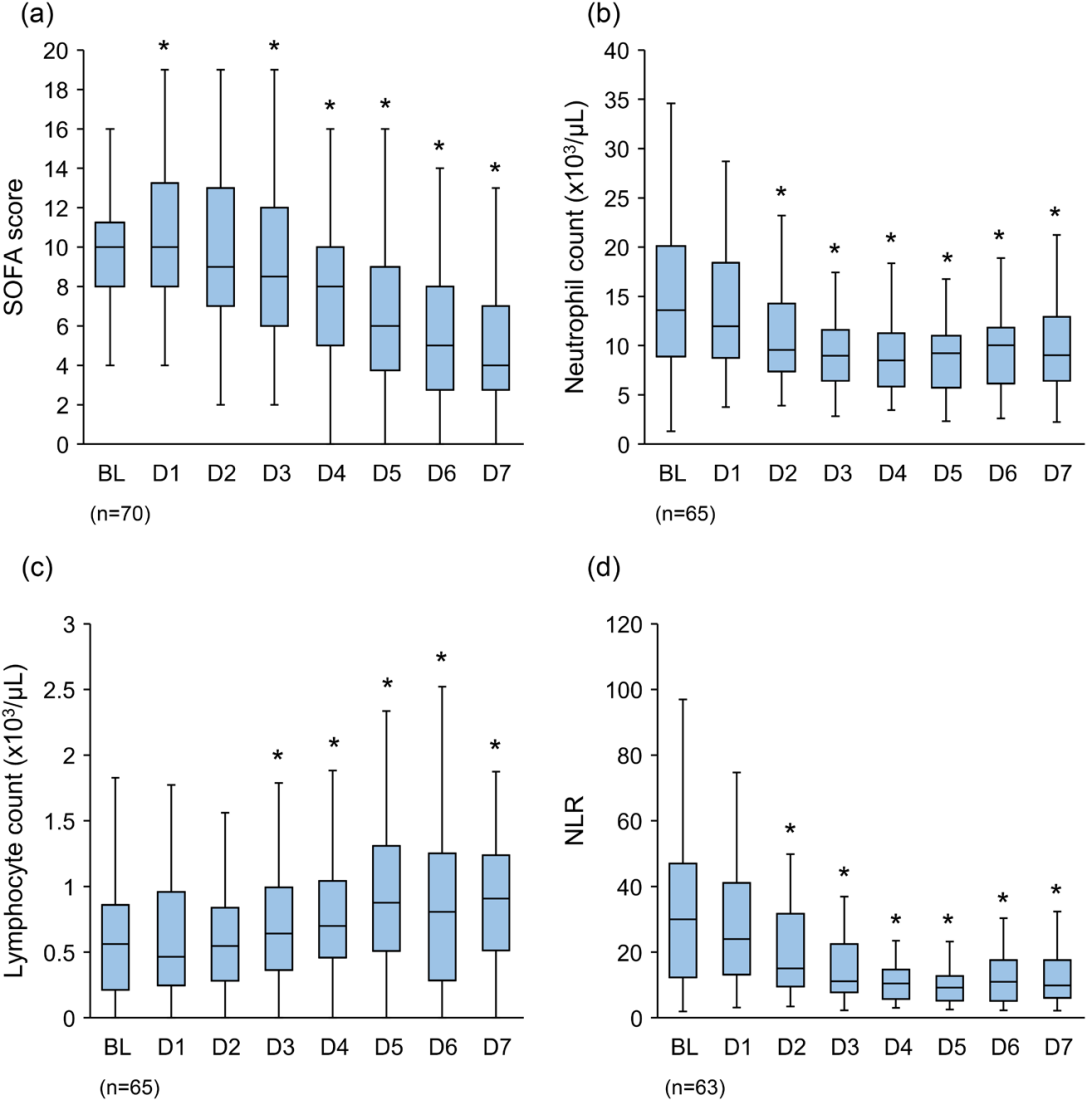
Changes in sequential organ failure assessment score (a), neutrophil count (b), lymphocyte count (c) and NLR (d) from baseline to Day 7. Analysis was conducted in a patient group that received G‐1 direct hemoperfusion at least once and for whom efficacy evaluation was possible. Asterisks indicate statistical significance (*p* < 0.05), and *p* values were calculated using the Wilcoxon signed‐rank test to test for changes from baseline. NLR: Neutrophil‐to‐lymphocyte ratio, BL: Baseline, DX: Day X. [Color figure can be viewed at wileyonlinelibrary.com]

A significant decrease in the SOFA score was observed from baseline to Day 7; the delta SOFA score on Day 7 was −5 (−7, −3) (*p* < 0.01) (Table 1, Figure 1). Of the 70 used for the SOFA score, 2 died after Day 7.

#### 3.4.2 Leukocyte Adsorption Rate and Number Adsorbed to the G1‐DHP

The number of granulocytes adsorbed by a single use of G1‐DHP was 6.50 × 10^9^ (4.49 × 10^9^–7.66 × 10^9^), and the adsorption rate was 10.7% (6.2%–14.6%). The number of monocytes adsorbed was 0.12 × 10^9^ (0.00–0.34 × 10^9^), and the adsorption rate was 10.4% (0.0%–20.4%). The number of lymphocytes adsorbed was 0.01 × 10^9^ (0.00–0.24 × 10^9^), and the adsorption rate was 0.6% (0.0%–8.1%).

#### 3.4.3 Neutrophils, Lymphocytes, and NLR

The neutrophil count significantly decreased from a baseline of 13.58 × 10^3^/μL (8.53 × 10^3^–20.11 × 10^3^) to 9.34 × 10^3^/μL (6.44 × 10^3^–12.93 × 10^3^) on Day 7 (*p* < 0.01). Conversely, the lymphocyte count significantly increased from a baseline of 0.58 × 10^3^/μL (0.22 × 10^3^–0.85 × 10^3^) to 0.91 × 10^3^/μL (0.54 × 10^3^–1.24 × 10^3^) on Day 7 (*p* < 0.01) (Table 1, Figure 1). The NLR at baseline of 30.00 (12.28–47.00) significantly decreased to 11.15 (7.73–22.5) on Day 3 and significantly further decreased to 9.88 (6.05–17.60) on Day 7 (*p* < 0.01) (Figure 1). Of the 65 used for neutrophil count data, 2 died after Day 7.

### 3.5 Organ‐Specific SOFA Score

**FIGURE 2:**
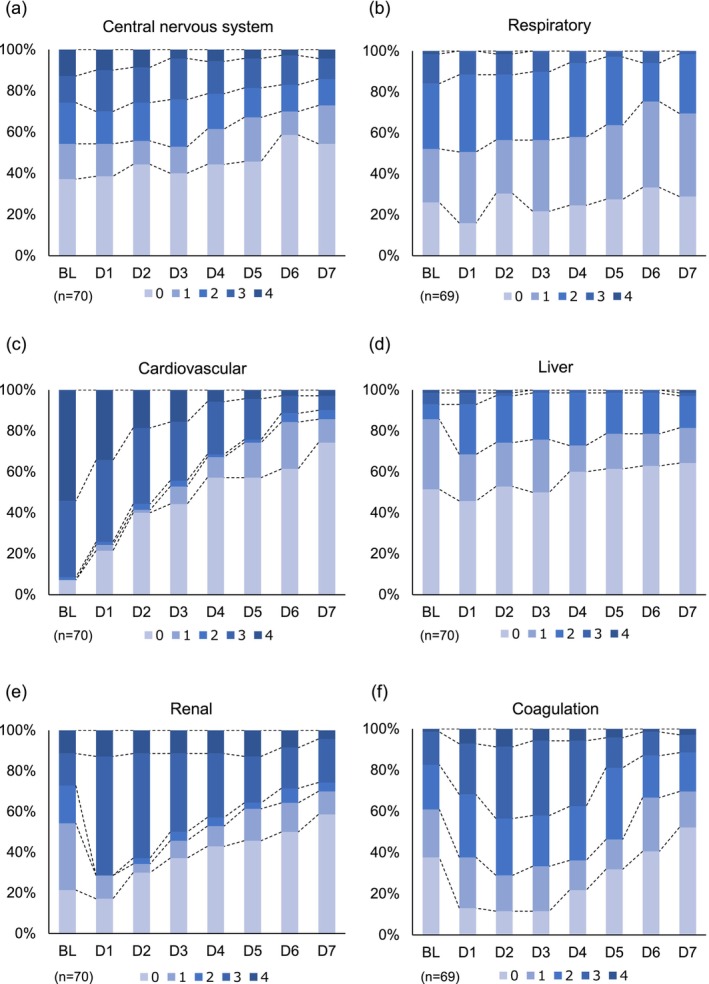
Changes in individual organ sequential organ failure assessment scores from baseline to Day 7. Analysis was conducted in a patient group that received G‐1 direct hemoperfusion at least once and for whom efficacy evaluation was possible. Frequency and percentage were calculated for each measurement point for the central nervous system score (a), respiratory score (b), cardiovascular score (c), liver score (d), renal score (e), and coagulation score (f) from baseline to Day 7 and shown in stacked bar charts. BL: Baseline, DX: Day X. [Color figure can be viewed at wileyonlinelibrary.com]

The changes in the organ‐specific SOFA scores from baseline to Day 7 showed a significant decrease in the central nervous system (−0.6 ± 1.50) (*p* < 0.01), respiratory (−0.4 ± 1.23) (*p* < 0.05), cardiovascular (−2.8 ± 1.34) (*p* < 0.01), renal (−0.6 ± 1.37) (*p* < 0.01), and coagulation (−0.3 ± 1.20) (*p* < 0.05) organs. The degree of improvement in organ dysfunction (proportion of a zero score at baseline and on Day 7) was greater than that in other organs, with a cardiovascular improvement from 10% to 70% and a renal improvement from 20% to 50% (Figure 2). There were no changes in the liver score.

The delta SOFA score showed a significant decrease in patients with concomitant CRRT (−3.52 ± 4.55) and without concomitant CRRT (−5.72 ± 3.35) (both *p* < 0.01). The change in renal SOFA score also showed a significant decrease in patients with concomitant CRRT (−0.65 ± 1.47) and without concomitant CRRT (−0.59 ± 1.28) (both *p* < 0.01).

### 3.6 Organ‐Specific Indicators

The MAP significantly increased from baseline to Day 3 (*p* < 0.05). The norepinephrine equivalent vasopressor dosage significantly decreased to 0.000 μg/kg/min by Day 7 (*p* < 0.01). The KDIGO stage showed a significant change from 3 (2–3) to 1 (0–3) (*p* < 0.01) from baseline to Day 7. Among the 78 patients who could be evaluated, the number of patients diagnosed with AKI decreased from 69 to 40 (*p* < 0.01). Serum creatinine significantly decreased from 1.67 mg/dL (1.18 mg/dL–2.91 mg/dL) to 0.90 mg/dL (0.59 mg/dL–1.56 mg/dL) (*p* < 0.01). The hourly urine volume, when divided by the KDIGO urine volume criteria on Day 1, was 89.9 mL/h (54.8 mL/h–174.7 mL/h) and decreased to 74.5 mL/h (48.0 mL/h–91.2 mL/h) in the 0–1 point group (46 patients) on Day 7, whereas in the 2–3 point group (20 patients), it significantly increased from 15.7 mL/h (4.4 mL/h–25.8 mL/h) to 56.4 mL/h (13.3 mL/h–122.6 mL/h) on Day 7 (*p* < 0.01). The PaO_2_/F_I_O_2_ ratio significantly increased from 284.0 (180.8–400.8) to 363.3 (287.7–410.6) on Day 7 (*p* < 0.01).

#### 3.6.1 Inflammatory Coagulation‐Related Tests

**FIGURE 3:**
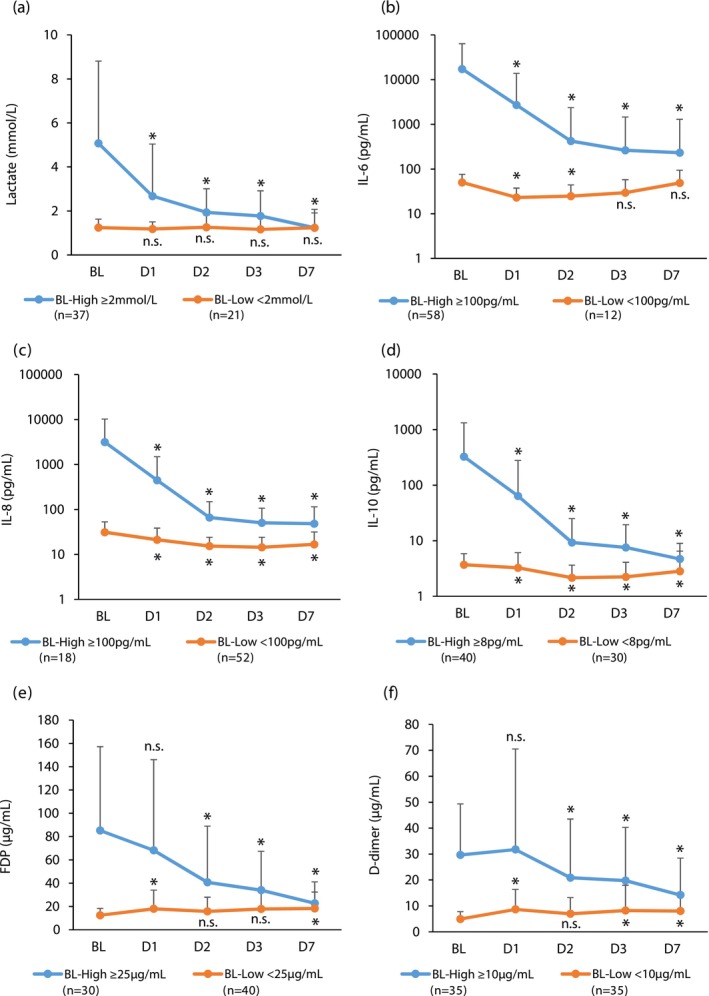
Changes in the lactate level (a), interleukin (IL)‐6 (b), IL‐8 (c), IL‐10 (d), fibrin degradation products (FDP) (e), D‐dimer (f) from baseline to Day 7. Analysis was conducted in a patient group that received G‐1 direct hemoperfusion at least once and for whom efficacy evaluation was possible. Asterisks indicate statistical significance (*p* < 0.05), and *p* values were calculated using the Wilcoxon signed‐rank test to test for changes from baseline. Patients were divided into high and low groups according to the initial levels of each measured parameter. Cut‐off values were 2 mmol/L for lactate, 100 pg/mL for IL‐6, and IL‐8, 8 pg/mL for IL‐10, 25 μg/mL for FDP, 10 μg/mL for D‐dimer. BL: Baseline, DX: Day X, n.s.: Not significant. [Color figure can be viewed at wileyonlinelibrary.com]

The lactate level significantly decreased from a baseline of 2.60 mmol/L (1.47 mmol/L–4.41 mmol/L) to 1.00 mmol/L (0.86 mmol/L–1.31 mmol/L) on Day 7 (*p* < 0.01) (Table 1). Of the 48 patients with DIC (a score ≥ 4), the number of patients with a high lactate level (≥ 4 mmol/L) was 21.8% (17/78 patients) at baseline, which decreased to 2.9% (2/70 patients) on Day 3 and 1.7% (1/58 patients) on Day 7. The platelet count showed a significant increase on Day 7 (Table 1). Transitions from baseline to Day 7 for IL‐6, IL‐8, IL‐10, FDP, and D‐dimer, divided into high and low groups, showed that all indices in the high groups were significantly decreased (Figure 3). The HMGB‐1 level significantly decreased from a baseline of 3.20 ng/mL (2.20 ng/mL‐4.53 ng/mL) to 1.95 ng/mL (1.40 ng/mL‐2.83 ng/mL) on Day 7 (*p* < 0.01).

#### 3.6.2 Evaluation of Safety

Of the 82 patients in whom G1‐DHP was used, 77 were observed until Day 28, with an APACHE II score of 27. No deaths were observed in the group with a decrease in SOFA score ≥ 2 (62 patients), which was the target. In contrast, in the group with a decrease in SOFA score of < 2 points (15 patients), six (40.0%) died.

## 4 Discussion

The Adacolumn was developed as a medical device to control excessive inflammatory reactions of granulocytes and monocytes. It was approved for use in Japan in 1999 with the aim of inducing remission of ulcerative colitis. Since then, it has been used domestically and internationally in clinical settings. The implementation conditions for the Adacolumn during the treatment of ulcerative colitis are set at 30 mL/min for 60 min per session. However, because sepsis is characterized by a higher degree of acute inflammation and demands early multidisciplinary treatment, we aimed to process the total circulating blood volume (approximately 6000 mL) with one use of G1‐DHP. The blood flow rate and perfusion time were examined in a pig lipopolysaccharide inflammation model, and it was confirmed that there were no adverse changes to the leukocyte adsorption characteristics or biocompatibility under administration conditions of 30–60 mL/min for 2 h [14]. Based on these results, the blood flow rate and perfusion time in this study were set at 50 mL/min for 120 min.

**FIGURE 4:**
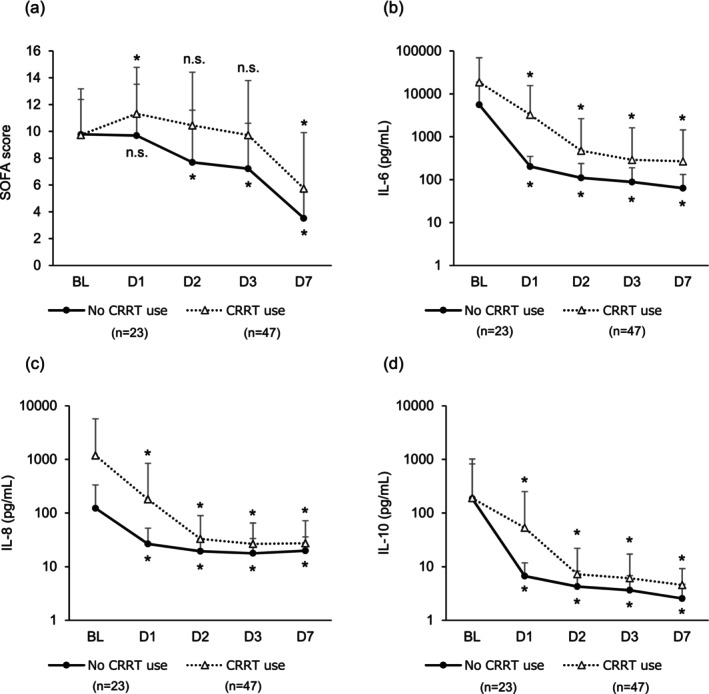
Changes in sequential organ failure assessment score (a), interleukin (IL)‐6 (b), IL‐8 (c), IL‐10 (d), from baseline to Day 7. Analysis was conducted in a patient group that received G‐1 direct hemoperfusion at least once and for whom efficacy evaluation was possible. Asterisks indicate statistical significance (*p* < 0.05), and *p* values were calculated using the Wilcoxon signed‐rank test to test for changes from baseline. Patients were divided into two groups: G1‐DHP with CRRT (*n* = 47) and G1‐DHP alone without CRRT (*n* = 23) during the first 7 days of treatment. BL: Baseline, DX: Day X, n.s.: Not significant.

In accordance with reports that a decrease of at least 2 in the SOFA score is associated with survival in patients with sepsis [20, 21], the goal of this study was a decrease of at least 2 in the SOFA score from baseline to Day 7. In this study, a significant decrease in the SOFA score of −5 (−7, −3) (*p* < 0.01) was observed from baseline to Day 7. As many patients in this study were treated with a membrane with cytokine adsorption characteristics, changes in cytokine concentrations and SOFA scores could be attributed to CRRT. Therefore, the patients were divided into two groups: G1‐DHP with CRRT (*n* = 47) and G1‐DHP without CRRT (*n* = 23) during the first 7 days of treatment. The G1‐DHP without CRRT group showed a significant decrease in IL‐6, IL‐8, and IL‐10 levels from baseline to Day 7 (all *p* < 0.01*). SOFA scores also significantly improved with and without CRRT (*p* < 0.01) (Figure 4). No deaths were observed in the 62 patients who had a decrease in the SOFA score ≥ 2, whereas six of 15 patients (40.0%) with a decrease in the SOFA score < 2 died. These results suggest that a decrease of 2 in the SOFA score may affect the outcome after 28 days.

In this study, we confirmed significant adsorption and removal of granulocytes and monocytes before and after the use of G1‐DHP. In contrast, the adsorption of lymphocytes was minimal. When blood passes through the G‐1, immunoglobulin (IgG) and inactivated complement‐3b (iC3b) in the blood are adsorbed onto cellulose acetate beads, and granulocytes and monocytes, which have Fc gamma receptors (FcγR) and complement receptor 3 for IgG and iC3b, are adsorbed and removed [22]. The number of neutrophils significantly decreased, and the number of lymphocytes significantly increased. The NLR significantly improved by Day 7 (Table 1, Figure 1). The number of lymphocytes often decreases during sepsis, and there have been reports that in non‐survivors, the decrease in lymphocyte count persists and does not recover beyond 0.7 × 10^3^/μL [23]. In this study, the baseline lymphocyte count was low at 0.58 × 10^3^/μL (0.22 × 10^3^/μL–0.85 × 10^3^/μL); however, it significantly increased to 0.91 × 10^3^/μL (0.54 × 10^3^/μL–1.24 × 10^3^/μL) on Day 7 (*p* < 0.01). The mechanism for the increase in lymphocyte count is unknown; however, it may be related to the fact that by Day 7 of G1‐DHP use, 87.2% of the patients no longer required norepinephrine, which has a side effect of reducing the lymphocyte count. The NLR is a similar indicator, and a recent meta‐analysis confirmed that the mortality rate of sepsis increased when the NLR cutoff was 10; the NLR decreases with improvement of sepsis [24], and the NLR of patients who die remains high [25]. The baseline NLR in this study was 30.00, which significantly decreased to 11.15 on Day 3 after the completion of five uses of G‐1 and was 9.88 on Day 7. G1‐DHP is the first treatment for sepsis that removes granulocytes, mainly neutrophils, and affects the NLR. This may have contributed to the improvement in survival rate due to the temporal increase in lymphocyte count.

Inflammatory mediators are produced during sepsis, and neutrophils adhere to the vascular endothelial cells and induce increased vascular permeability and vasodilation. As a result, blood pressure decreases owing to a relative decrease in the circulating blood volume [26]. In this study, the MAP increased by 6.1 mmHg from baseline to Day 3. Accordingly, in this study, when G1‐DHP was used at a norepinephrine administration rate of up to 0.1 μg/kg/min, administration was no longer necessary in 87.2% (68/78) of patients by Day 7.

Norepinephrine, which is administered to treat sepsis, is also known to decrease the number of lymphocytes in the blood, suppress immunity [27], impair microcirculation, and decrease organ blood flow [28]. It is possible that early discontinuation of norepinephrine contributes to recovery from immunodeficiency due to an increase in the lymphocyte count and recovery from organ dysfunction by improving microcirculation.

KDIGO stage improved on Day 3 (median 3 (2,3) to 3 (0,3), *p* < 0.01; mean 2.36 ± 1.01 to 1.84 ± 1.37, *p* < 0.01), and serum creatinine also improved significantly on Day 3 (1.67 mg/dL to 0.85 mg/dL; *p* < 0.01). Renal SOFA scores significantly improved with and without CRRT.

NGAL is a biomarker produced by renal tubular cells, and a high urinary NGAL level is associated with mortality. A recent meta‐analysis reported a cut‐off level of 580 ng/mL for risk detection [29]. In this study, the baseline urinary NGAL level was high (782 ng/mL). Few studies have examined the progression of the urinary NGAL level; however, prolonged elevation (baseline: 552 ng/mL to Day 3: 751 ng/mL) has been reported in septic AKI [30]. Proximal tubular injury during AKI has also been shown to be caused by neutrophil infiltration into the kidney [31]. In this study, 69 patients (88.5%) were diagnosed with AKI, which improved to 40 (52.6%) by Day 7. These results suggest that G1‐DHP may mitigate tubular injury. Based on these results, G1‐DHP may be considered for the treatment of septic AKI.

Although non‐survivors of sepsis have a prolonged high IL‐6 level, the high IL‐6 group in this study showed a decrease of approximately one‐tenth from baseline to Day 1.

The mortality rate of patients with septic DIC is high, and those with a high lactate level are at high risk. In this study, the DIC score significantly improved from 4 (3–5) to 2 (1–4). The 28‐day mortality rate of patients with septic DIC was 4.3% (2/47 cases), which is lower than that reported by Hasegawa et al. [32]. Seventeen patients with DIC and a high blood lactate level (> 4 mmol/L) [32] decreased to two patients on Day 3 and one patient on Day 7. G1‐DHP improved laboratory data on massive inflammation, coagulation abnormalities, and microcirculatory disturbances relative to each other. It is known that the platelet count decreases during treatment of sepsis due to inflammation, transfusion, and extracorporeal circulation. In this study, a decrease in the platelet count was also observed over the course of 3 days. These cases should be noted as they may represent false‐positive DIC diagnoses.

It was difficult to determine the optimal timing for G1‐DHP treatment. The study showed that G1‐DHP can improve laboratory indices when applied before or after the borderline that separates mortality and survival, such as a norepinephrine dose > 0.1 μg/kg/min, KDIGO stage 3, lymphocyte count < 1000/μL, NLR > 10, and lactate level > 2 mmol/L. To our knowledge, this is the first study to demonstrate the safety and experimental efficacy of G1‐DHP in patients with sepsis. Neutrophils may have a significant role in the pathophysiology of sepsis, and our strategy of using a granulocyte adsorption column as a “decoy organ” to attract neutrophils may have contributed to the mitigation of organ dysfunction.

This study has some limitations. First, owing to its single design, the evaluation of effectiveness is limited. Sepsis is a highly heterogeneous disease, and it is necessary to evaluate effects in specific subtypes where effects are expected. However, there was no information regarding the effective subtypes of G1‐DHP at the time of study planning. We aimed to exclude mild cases by limiting the severity to patients with sepsis with an APACHE II score of 17–34. Additionally, taking time for random allocation could delay the initiation of standard treatments, such as antibiotics and fluids, and could potentially delay the timing of G1‐DHP treatment. Therefore, this study was a single trial that focused on the safety evaluation and experimental effectiveness of G1‐DHP. It was not possible to confirm that the improvements in SOFA scores and NLR occurred without G1‐DHP because there was no control group. The 28‐day survival rate in this study was 92%; therefore it is natural that patient SOFA scores should improve, thus the improvement in SOFA scores in this study is not evidence of the efficacy of G1‐DHP. It may be necessary, in future studies, to focus on more severely ill patients or with specific organ damage to demonstrate the efficacy of G1‐DHP. Second, we were unable to demonstrate the effectiveness of G1‐DHP alone, as it would be necessary to limit treatments, such as steroid therapy and blood purification methods, that affect the immune system. However, limiting the treatment of severely ill patients could be disadvantageous; therefore, we decided to evaluate this comprehensively by adding G1‐DHP to the standard treatment. In this study, organ damage indices that would theoretically be improved by removing granulocytes were examined. However, a clinical effect specific to G1‐DHP cannot be confirmed because there was no control group. Third, we could not establish the mechanisms underlying the efficacy of G1‐DHP. The function of white blood cells can be clarified by examining their surface antigens. However, we judged that it was not realistic to perform cell surface antigen analysis immediately after blood collection at each facility. Fourth, we attempted to clarify the optimal timing for G1‐DHP by analyzing the patient backgrounds but were unable to determine it. However, we believe that we have provided useful information that will be used in future studies. Future studies may reveal the specific efficacy of G1‐DHP by comparing it to a control group, and analyses of leukocyte surface antigen markers may provide additional insights into the mechanism underlying the efficacy.

## 5 Conclusions

G1‐DHP adsorbed and removed granulocytes, mainly neutrophils, from patients with sepsis. We confirmed that G1‐DHP could be safely used in addition to conventional treatments for sepsis. Further studies using different inclusion criteria and/or treatment protocols are required.

## Author Contributions

Osamu Nishida was mainly responsible for the concept/design, interpreting the data, and writing the manuscript. Kazuhiro Moriyama contributed to data analysis and the writing. All authors contributed to the data collection. All the authors have read and approved the final version of the manuscript.

## Ethics Statement

This study was conducted in accordance with the Declaration of Helsinki, the Japanese Pharmaceutical and Medical Device Act, and Good Clinical Practice guidelines (GCP). The study was approved by the ethics committee of each institution and the Pharmaceuticals and Medical Devices Agency and was performed according to the guidelines of the relevant institutional review boards.

## Consent

Written informed consent was obtained from all patients or their parents/legal guardians/next of kin.

## Conflicts of Interest

Kazuhiro Moriyama is a member of the Endowed Chair of JIMRO Co. Ltd. The other authors declare no conflicts of interest.
